# MSC stimulate ovarian tumor growth during intercellular communication but reduce tumorigenicity after fusion with ovarian cancer cells

**DOI:** 10.1186/s12964-018-0279-1

**Published:** 2018-10-13

**Authors:** Catharina Melzer, Juliane von der Ohe, Ralf Hass

**Affiliations:** Biochemistry and Tumor Biology Lab, Department of Obstetrics and Gynecology (OE 6410), Hannover Medical School, Carl-Neuberg-Str. 1, D –30625 Hannover, Germany

**Keywords:** Mesenchymal stem cells, Breast and ovarian cancer, Tumor microenvironment

## Abstract

The tumor microenvironment enables important cellular interactions between cancer cells and recruited adjacent populations including mesenchymal stroma/stem cells (MSC). In vivo cellular interactions of primary human MSC in co-culture with human SK-OV-3 ovarian cancer cells revealed an increased tumor growth as compared to mono-cultures of the ovarian cancer cells. Moreover, the presence of MSC stimulated formation of liver metastases. Further interactions of MSC with the ovarian cancer cells resulted in the formation of hybrid cells by cell fusion. Isolation and single cell cloning of these hybrid cells revealed two differentially fused ovarian cancer cell populations termed SK-hyb1 and SK-hyb2. RNA microarray analysis demonstrated expression profiles from both parental partners whereby SK-hyb1 were attributed with more SK-OV-3 like properties and SK-hyb2 cells displayed more similarities to MSC. Both ovarian cancer hybrid populations exhibited reduced proliferative capacity compared to the parental SK-OV-3 cells. Moreover, the fused populations failed to develop tumors in NODscid mice. Together, these data suggested certain stimulatory effects on ovarian tumor growth in the presence of MSC. Conversely, fusion of MSC with SK-OV-3 cells contributed to the generation of new cancer hybrid populations displaying a significantly reduced tumorigenicity.

**Electronic supplementary material:**

The online version of this article (10.1186/s12964-018-0279-1) contains supplementary material, which is available to authorized users.

## Background

One of the most lethal gynecologic malignancies is caused by ovarian cancer. The majority of epithelial ovarian cancers is categorized into two types. Type I ovarian tumors include low-grade serous, endometrioid, clear cell and mucinous carcinomas carrying gene mutations of KRAS, BRAF, ERBB2, PTEN, CTNNB1, and PIK3CA among others which appear clinically indolent. Conversely, type II tumors often display genetic instabilities with a high frequency of TP53 mutations and cyclin E1 amplifications and are characterized as high-grade serous, high-grade endometrioid or undifferentiated carcinomas [1, 2]. Moreover, malignant mixed mesodermal tumors (carcinosarcomas) with papillary, glandular, and solid patterns are predominantly observed in advanced ovarian tumor stages and display highly aggressive cancer cells [3–5].

Development and progression of ovarian cancer represents a complex multistep cascade during malignant conversion and interactions with adjacent cell types in the tumor microenvironment including mesenchymal stroma/stem-like cells (MSC) [6]. MSC preferentially reside in perivascular niches of nearly all kinds of human tissues [7, 8]. Despite functional differences according to their tissue-specific origins, heterogenic MSC populations share distinct surface marker expressions such as CD73, CD90, and CD105, and they maintain the capability to differentiate at least along certain phenotypes of the mesodermal lineage [9–12]. Moreover, MSC contribute to regulate stem cell homeostasis, migrate towards damaged or injured tissues to utilize repair processes [13], support angiogenesis [14] and modulate immune cell functions [15].

According to this multi-functional plasticity, intracellular expression levels of several miRs contribute to alter the MSC state of activation and susceptibility [16]. Consequently, MSC are considered cellular all-round supporters and exhibit a significant sensitivity to mutual extracellular signaling with normal and carcinoma cell populations [17–20]. Distinct functions within this unique panel of MSC biodiversity can be triggered by alterations of the microenvironment such as the threshold of cytokines/chemokines to induce MSC adherence [21], changes in the extracellular matrix composition, and determination of a direct cell-to-cell contact.

Although MSC and their multi-functionality play an important role in combination with several different types of carcinoma cells such as breast and ovarian cancer cells, little is known about the mechanisms involved and resulting effects can be controversial. Thus, cellular interactions of MSC can develop opposite effects in ovarian cancer cells, whereby the underlying mechanisms remain unclear. Previous work has demonstrated that MSC extracts derived from either MSC lysates or supernatants inhibit cell growth of a variety of carcinoma cell lines including breast, ovarian, and osteosarcoma cells [22]. Conversely, human MSC were suggested to promote ovarian cancer growth and support proliferation and survival [23]. In fact, intercellular communication of MSC with different carcinoma cells is associated with mutual functional alteration including enhanced tumor growth and elevated metastatic potential [24, 25]. Moreover, studies in breast cancer cells revealed that interaction with MSC can also generate new cancer hybrid populations by cellular fusion [26–28].

To address this controversial issue, the present study in ovarian carcinoma models offers some new data to potential tumor-supportive and tumor-inhibitory effects of MSC. The results demonstrate that initial ovarian tumor growth is enhanced by cellular communication in the presence of MSC. However, tight interaction and subsequent fusion of MSC with ovarian cancer cells generate new populations displaying reduced tumorigenicity.

## Materials and methods

### Cell culture

Isolation of primary human MSC was performed from umbilical cord explant cultures as reported previously [29] and cultured in αMEM (Sigma Chemie GmbH, Steinheim, Germany) supplemented with 10% of allogeneic human AB-serum (commercially obtained from blood bank, University Campus Lübeck, Germany), 100 U/ml penicillin, 100 μg/ml streptomycin and 2 mM L-glutamine (Sigma). The use of primary human mesenchymal stem cells following explant culture from umbilical cord tissue has been approved by the Ethics Committee of Hannover Medical School, Project #443 on February 26th, 2009, respectively, and informed written consent was obtained from the patient.

MSC were subcultured following accutase (Sigma) treatment for 3 min at 37 °C. MSC from different donors and passages (MSC060616 P5 and MSC081113 P6) were used in the experiments.

Human SK-OV-3 ovarian cancer cells (ATCC® #HTB-77TM) were commercially obtained in P25 from the ATCC, Manassas, VA, USA. These cells were originally established from the malignant ascites of a patient with progressive adenocarcinoma of the ovary. SK-OV-3 cells were cultivated at about 1,750 cells/cm^2^ in RPMI 1640 supplemented with 10% (*v*/v) fetal calf serum, 100 U/ml L-glutamine, 100 U/ml penicillin and 100 μg/ml streptomycin. Subculture was performed by trypsin/EDTA (Biochrom GmbH, Berlin, Germany) treatment for 5 min at 37 °C.

### Cell line testing and authentication

All cells were tested for mycoplasma by the luminometric MycoAlert Plus mycoplasma detection kit (Lonza Inc., Rockland, ME, USA) according to the manufacturer’s recommendations. Cell line authentication was performed by short tandem repeat (STR) fragment analysis using the GenomeLab human STR primer set (Beckman Coulter Inc., Fullerton, CA, USA). The STR pattern of SK-OV-3 ovarian cancer cells was confirmed in previous work [30] according to the STR database provided by the ATCC, Manassas, VA, USA.

### Co-culture of MSC with human ovarian cancer cells and isolation of hybrid cells

For co-culture experiments with MSC populations, SK-OV-3 ovarian cancer cells were previously adapted to MSC culture medium. In order to distinguish the different cell types and newly formed hybrid cells within the in vitro co-culture, MSC and ovarian cancer cells have been transduced with a 3rd generation lentiviral SIN vector containing the eGFP and the mcherry gene, respectively, as indicated in previous work [25]. Following 7d co-culture of MSC081113^GFP^ P6 with SK-OV-3^cherry^ P90 (cell ratio 60:40 at a density of 2,000 cells/cm^2^) in MSC medium, a total amount of 5.7 × 10^7^ co-cultured cells was subjected to repeated separation by fluorescence-activated cell sorting (FACS). The first separation step yielded 2.4 × 10^4^ cells identified as double positive for mcherry and GFP. However, this population still contained false positive and doublet cells and therefore, a second FACS separation was performed with this population resulting in 150 double positive for mcherry and GFP. These hybrid cells were cultured as single cell per well in 96-well microtiter plates (Nunc) and from these 150 clones, two initially proliferating hybrid clones of MSC/SK-OV-3 co-culture termed SK-hyb1 and SK-hyb2 were isolated and further analyzed.

### In vivo experiments

Animal research using NOD/scid mice was carried out by following the internationally recognized guidelines on animal welfare and has been approved by the institutional licensing committee ref. # 33.19–42502–04-15/1992 on Dec. 18th, 2015.

About 4.5 × 10^5^ GFP-labeled SK-OV-3^GFP^ cells as both mono- or co-culture (together with 4.5 × 10^5^ MSC060616) were injected subcutaneously into 5 animals of 5 to 6 weeks old female NOD/scid mice, respectively. After 38d post injection, all 10 mice had developed subcutaneous tumors and the animals were sacrificed by cervical dislocation. Primary tumor tissues were dissected under UV light, weighted, washed in PBS, and subsequently cultured in vitro for explant culture of the tumor cells. Organs were also dissected from the mice and thin sections were analyzed by fluorescence microscopy for presence and accumulation of metastatic cells.

In a separate set of experiments, 4.5 × 10^5^ SK-hyb1, and SK-hyb2 cells were injected subcutaneously into NOD/scid mice, respectively, and tumor development was compared to that of 4.5 × 10^5^ SK-OV-3^GFP^ parental control cells.

### Transcript analysis by RT-PCR

Total RNA was isolated using RNeasy Mini Kit (Qiagen, Hilden, Germany) according to the manufacturer’s instructions. One μg RNA was reverse transcribed into cDNA using 500 μM of dNTP (R0193), 5 μM Oligo(dT)18 primer (S0132), 5 μM Random Hexan primer (S0142), 1 U RiboLockTM RNase Inhibitor (E00381) and 5 U RevertAidTM M-MuLV Reverse Transcriptase (EP0441) in the supplied reaction buffer (all reagents from Thermo Scientific, Schwerte, Germany). The cDNA reactions were performed for 10 min/25 °C, 1 h/37 °C and stopped at 72 °C for 10 min. cDNA (2.5 μl) was used as a template with following specific primers (customized by Eurofins, MWG GmbH, Ebersberg, Germany) as described previously [27, 28, 31].

PCR reactions included 0.2 μM of each primer, 200 μM of dNTP (R0193, Thermo Scientific) and 0.03 U One Taq Hot Start DNA polymerase (New England Biolabs GmbH, Frankfurt am Main, Germany) in the supplied reaction buffer. PCR cycling conditions were performed 30s at 94 °C, 1 min at 60 °C and 68 °C for 1 min respectively, including an initial 30s denaturation step at 94 °C and a final 5 min extension step at 68 °C (35 cycles). Aliquots of 25 μl of each RT-PCR product were separated on a 2% agarose gel including the standard GeneRuler 100 bp DNA Ladder (Thermo Scientific) and visualized by GelRedTM (Biotium Inc., Hayward, CA, US) staining.

### Microarray-based mRNA expression analysis (single color mode)

RNA microarray analysis was performed according to the previously described details [27]. The parental cells SK-OV-3^cherry^ and MSC081113^GFP^ were investigated and likewise the resulting two hybrid populations SK-hyb1 and SK-hyb2. Alterations in transcript levels were compared between parental cells and hybrid cells displaying a more than 2-fold difference in gene expression. Microarray data are uploaded at NCBI database with accession no. GSE117411.

### Cell cycle analysis

The cell cycle analysis in the ovarian cancer cells, MSC, and SK-hybrid cells was performed as described previously [32]. Briefly, 10^5^ cells were fixed in 70% (*v*/v) ice-cold ethanol at 4 °C for 24 h. Thereafter, the fixed cells were stained with propidium-iodide for 30 min at room temperature. The samples were then measured in a FACSCalibur (BD Biosciences, Singapore) flow cytometer and analyzed using the FlowJo V10 software.

### Cytotoxicity measurements of explant tumor cultures by fluoroscan assay

The proliferative capacity in the absence and presence of different chemotherapeutic compounds was tested in steady-state SK-OV-3^GFP^ in vitro cell cultures compared to ex vivo explant cultures from SK-OV-3^GFP^-induced NODscid mouse tumors and MSC + SK-OV-3^GFP^ co-cultured tumors. Fluorescence measurement using the fluoroscan assay was performed as previously described [33]. Briefly, 3,000 cells/well were seeded with standard culture medium (100 μl/well) in flat bottom 96-well plates (Nunc/ThermoFischer Scientific, Roskilde, Denmark) and incubated overnight to allow attachment. Thereafter, 100 μl of culture medium with drug solvent was added to the cells as a control and in further wells 100 μl of culture medium containing different chemotherapeutics were added to the cells. Following incubation for up to 72 h, the medium was removed and the cells were lysed with 5% (*w*/*v*) SDS. Afterwards, the fluorescence intensities of GFP in the cell homogenate which corresponded to the appropriate cell number of cancer cells was measured at excitation 485 nm/emission 520 nm using the Fluoroscan Ascent Fl (ThermoFisher Scientific).

## Results

**Fig. 1:**
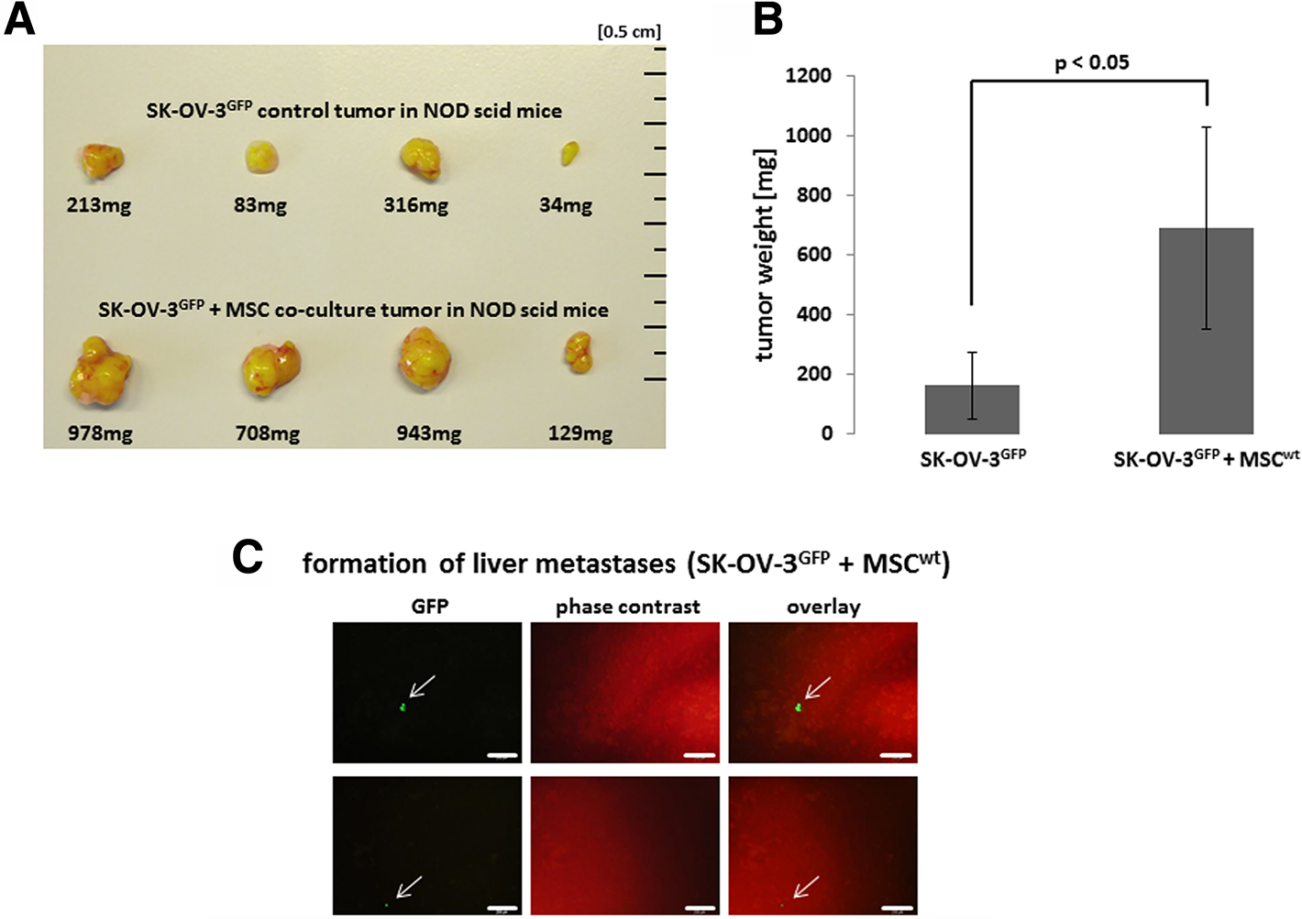
**a** NODscid mouse tumors were derived following injection of human SK-OV-3^GFP^ ovarian cancer cells in comparison to a co-injection of SK-OV-3^GFP^ and MSC060616^wt^ in MSC medium. **b** Tumor weight of SK-OV-3^GFP^-induced mouse tumors was compared to tumors after co-injection. Significance (p) was calculated by the mean ± s.d. (*n* = 4) using student’s t-test. **c** Formation of distant metastases was detected by GFP-fluorescence evaluation in thin sections from organ tissues and exemplary pictures from tissue thin section phase contrast/fluorescence microscopy of liver metastases are documented (arrows). Bars represent 200 μm

In vivo co-injection of human SK-OV-3 ovarian cancer cells with human MSC exhibited a significantly enhanced tumor growth in NODscid mice (Fig. 1a). Tumor weight was increased by 4.2-fold in the presence of MSC (Fig. 1b). All organs were tested for potential metastases whereby lung, spleen, heart, kidney, and brain revealed no detectable metastatic cells. However, MSC-mediated enhanced tumor development was associated with appearance of GFP-labeled tumor cells in the liver in contrast to SK-OV-3 cells-induced tumors without MSC co-injection (Fig. 1c).

**Fig. 2:**
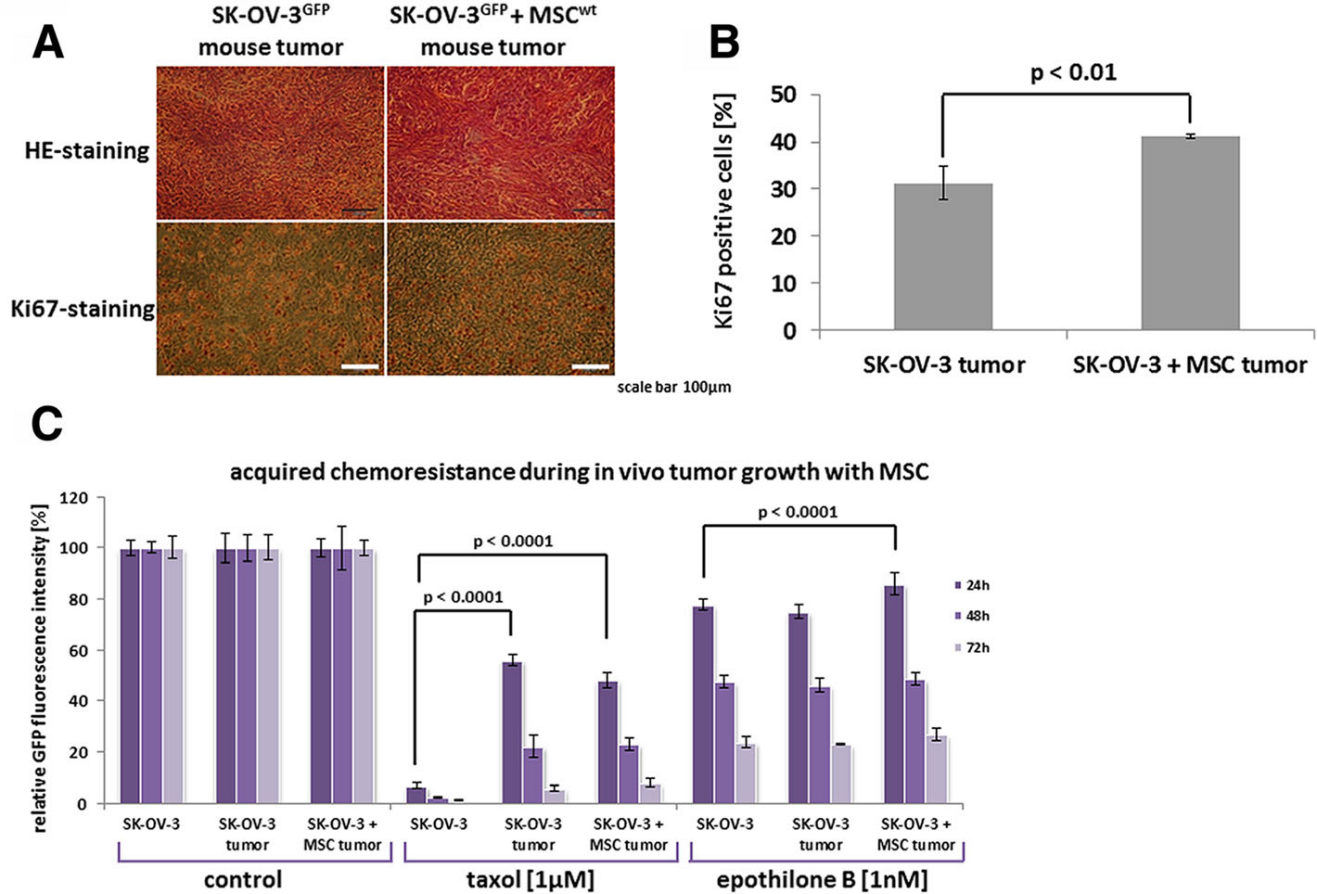
**a** Tissue sections (4 μm thick) of SK-OV-3^GFP^ control tumors and SK-OV-3^GFP^/MSC060616^wt^ co-injected tumors were stained for HE and Ki67 following fixation in paraformaldehyde. A representative selection of tumor areas is shown. (Bars = 100 μm). **b** Ki67 positive cells in the histopathologic tissue samples were counted and the percentage was calculated in SK-OV-3^GFP^ control tumors compared to SK-OV-3^GFP^/MSC060616^wt^ co-injected tumors. Significance (p) was calculated by the mean ± s.d. using student’s t-test. **c** Chemotherapeutic response was tested in SK-OV-3^GFP^ control cells and cell populations derived from tumor explant cultures of SK-OV-3^GFP^ tumors and SK-OV-3^GFP^/MSC060616^wt^ co-injected tumors. Relative proliferative capacity from steady state SK-OV-3^GFP^ cells and the mouse tumor-derived explant populations was evaluated in a fluoroscan assay following exposure to appropriate concentrations of different chemotherapeutics for 24 h up to 72 h. Data were calculated as the mean ± s.d. (*n* = 5) whereby fluorescence values were set = 100% for the corresponding cells in control medium. Significance (p) was calculated using ANOVA followed by Dunnett’s multiple comparisons test

Histopathological evaluation of the mouse tumors in 4 μm thick and formalin-fixed tissue sections by hematoxylin/eosin (HE) staining demonstrated various filament structures in MSC co-injected tumors which were not observed in SK-OV-3-induced tumors alone (Fig. 2a, upper panel). Staining of the tumor tissues with the proliferation marker Ki67 revealed 41.2 ± 0.5% positive cells (*n* = 3) in MSC + SK-OV-3 co-injected tumors as compared to 31.3 ± 3.6% in SK-OV-3-induced tumors alone (Fig. 2a, lower panel, Fig. 2b).

Ex vivo explant culture was performed with SK-OV-3-induced tumors and MSC + SK-OV-3 co-injected tumors to obtain corresponding primary cultures for chemotherapeutic comparison to the steady-state SK-OV-3 in vitro cell culture. While exposure to 1 μM taxol reduced the viability of SK-OV-3 in vitro cell culture down to 6.7 + 1.1% after 24 h, both ex vivo explant tumor cultures were significantly less sensitive with remaining 56.1 + 2.2% viable SK-OV-3 and 48.2 + 3.1% viable co-injected tumor explants after 24 h. Similar effects were observed after 48 h and 72 h. Of interest, epothilone B (epo B), a tubulin inhibitor similar to taxol, demonstrated less differences between SK-OV-3 in vitro cell culture and the two ex vivo explant tumor cultures although a significantly reduced sensitivity was still observed in the tumor cultures (Fig. 2c). Treatment with 1 μM carboplatin revealed relative resistance of all three cell populations after 72 h (Additional file 1: Figure S1). Higher concentration of 10 μM carboplatin progressively decreased the viability of steady-state SK-OV-3 control tumor cells, however, the tumor explant cultures remained unaffected (Additional file 1: Figure S1). Moreover, exposure to 100 μM carboplatin demonstrated cytotoxity in all populations with fewer effects in the explant cultures (Additional file 1: Figure S1). Together, these data further substantiated a reduced chemosensitivity in the ex vivo cultured tumors.

SK-OV-3-derived tumors and MSC co-injected tumors from three mice were examined for expression of MSC markers in comparison to their corresponding explant cell cultures, respectively. All tissue samples and cell populations expressed the MSC markers CD44, CD73, and CD105 with unaltered GAPDH levels as a loading control. However, little if any CD90 expression was detectable in either sample (Additional file 2: Figure S2).

**Fig. 3:**
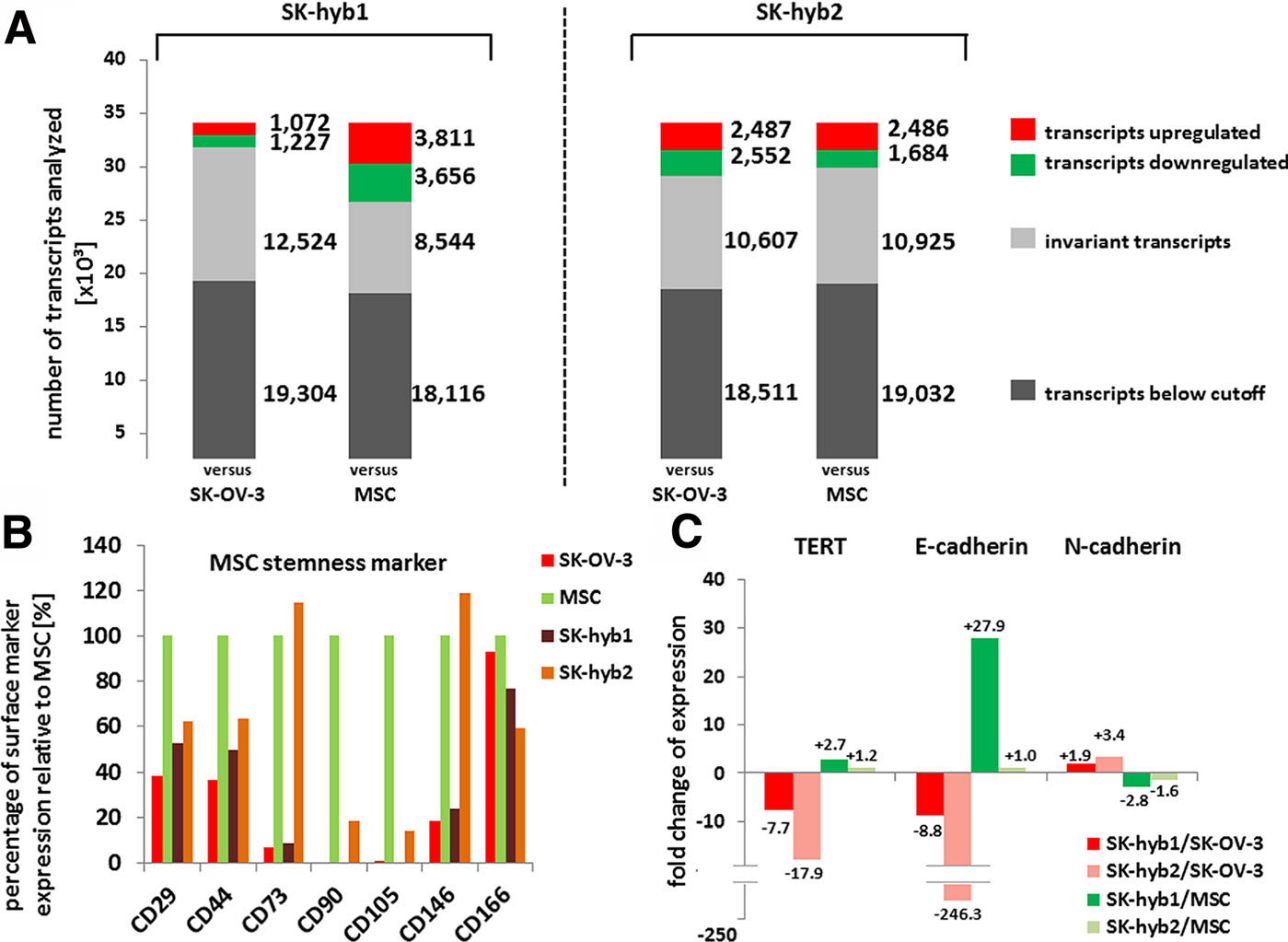
**a** Following RNA microarray analysis, changes in gene expression levels were quantified in SK-hyb1 (left panel) and SK-hyb2 cells (right panel) versus the corresponding parental cell populations SK-OV-3^cherry^ P90 and MSC081113^GFP^ P6, respectively. Changes in transcript levels of more than 2-fold were considered as up- or down-regulated. The entire set of microarray data is stored with the accession no. GSE117411 at the NCBI-GEO database. **b** Relative expression analysis based on the RNA microarray data of some characteristic mesenchymal stem-like markers was calculated for SK-OV-3 cells and the hybrid populations SK-hyb1 and SK-hyb2. For relative evaluations the expression levels of MSC were used as a control (set to 100%). **c** Functional changes of SK-hyb1 and SK-hyb2 cells by altered expression of telomerase (TERT) and cadherins when compared to the parental cell populations SK-OV-3^cherry^ P90 and MSC081113^GFP^ P6, respectively. Numbers indicate the fold changes of increased (+) or decreased (−) transcript levels

During interaction of SK-OV-3^cherry^ ovarian cancer cells with different MSC^GFP^ the generation of hybrid populations was observed displaying the fluorescence of both partners (Additional file 3: Figure S3). While cell fusion represents a rare event in general, most hybrid cells died during the post-fusion selection process and two initially proliferating clones were obtained yielding about 1 hybrid cell clone per 2.85 × 10^7^ cells in co-culture. These findings substantiate previous observations [25] and the two different hybrid cell clones SK-hyb1 and SK-hyb2 were isolated and further characterized. RNA microarray analysis of these SK-hyb1 and SK-hyb2 cells was performed in comparison to the parental populations SK-OV-3^cherry^ and MSC081113^GFP^, respectively (Fig. 3a). While the majority of genes referred to invariant and below cutoff transcripts, SK-hyb1 cells displayed a more SK-OV-3-like phenotype with 1,072 up- and 1,227 down-regulated transcripts, however, about 3,656 to 3,811 genes were differentially expressed compared to the parental MSC (Fig. 3a, left panel). Conversely, SK-hyb2 cells exhibited slightly less changes in mRNA expression levels versus MSC than versus SK-OV-3 cells indicating a more MSC-like phenotype (Fig. 3a, right panel).

These results were substantiated for the expression of different cell surface markers associated with MSC stemness. In particular, the markers CD73, CD90, CD105 and CD146 revealed a similar expression pattern for SK-OV-3 and SK-hyb1 on one side and for parental MSC and SK-hyb2 on the other side (Fig. 3b). Of interest, prominent expression of telomerase in SK-OV-3 cells was significantly reduced in the SK-hyb1 and SK-hyb2 cells and only slightly increased in relation to MSC (Fig. 3c). Moreover, E-cadherin was down-modulated by 8.8-fold in SK-hyb1 and by 246.3-fold in SK-hyb2 compared to SK-OV-3 cells, and up-regulated compared to MSC. Vice versa, N-cadherin was slightly up-regulated in the hybrid cells in relation to SK-OV-3 cells paralleled by a down-modulation compared to MSC (Fig. 3c). Again, the findings suggest increasing mesenchymal-like characteristics in both hybrid populations whereby SK-hyb2 cells displayed more similarities to the parental MSC as compared to SK-hyb1.

**Fig. 4:**
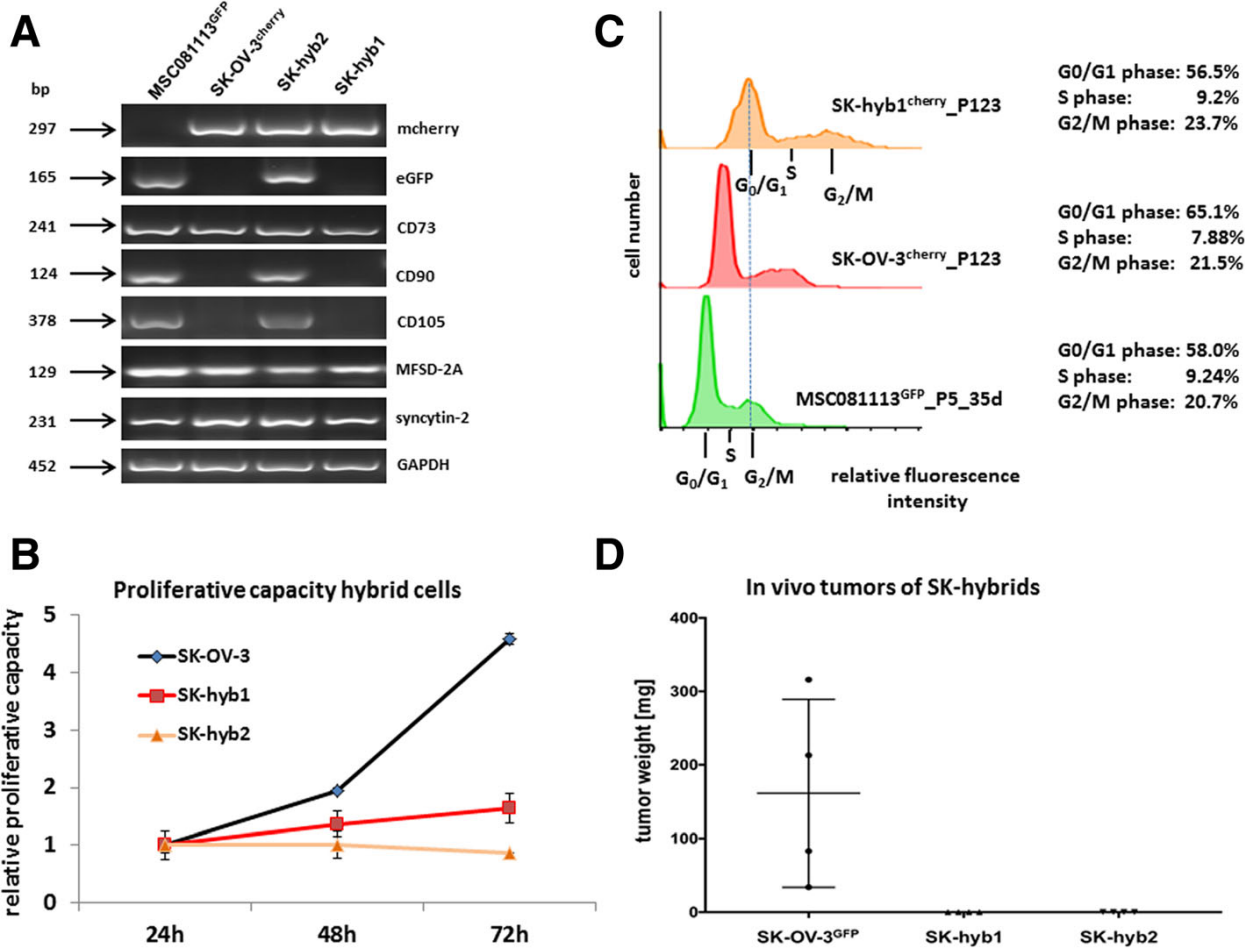
**a** PCR analysis was performed with mcherry, eGFP, MSC stem-like markers CD73, CD90, CD105, and fusion-associated factors syncytin-2 and MFSD-2A expression in the parental SK-OV-3^cherry^ and MSC^GFP^ populations as compared to the two hybrid populations SK-hyb1 and SK-hyb2, respectively. Unaltered GAPDH transcripts served as loading control. **b** Proliferative capacity of the parental SK-OV-3^cherry^ cells was compared to the SK-hyb1 and SK-hyb2 hybrid populations by cell counting of initially seeded 10^4^ cells/well in 24-well plates for 24 h up to 72 h, respectively. Data represent the mean + s.d. (*n* = 3). **c** Cell cycle analysis was performed in steady state SK-hyb1 cells and compared to the parental control populations SK-OV-3^cherry^ and MSC^GFP^ using the FlowJo V10 software. Cell cycle sub-populations of similar relative fluorescence intensities were matched by the dotted line indicating that the DNA content of MSC in G_2_/M corresponded to SK-hyb cells in G_0_/G_1_ phase. **d** Tumor weight of SK-OV-3^GFP^-induced tumors (*n* = 4) was measured following dissection of the solid subcutaneous primary tumors. No tumors were detectable after transplantation of either SK-hyb1 or SK-hyb2 cell populations

Hybrid cell formation was also supported by RT-PCR demonstrating simultaneous expression of both fluorescence genes in SK-hyb2 cells while the SK-hyb1 population lost the eGFP plasmid. mRNA transcripts of the MSC markers CD73, CD90, CD105 revealed low to undetectable levels of CD90 and CD105 in SK-OV-3 and SK-hyb1 cells. All four cell populations expressed the fusion-associated gene syncytin-2 and the corresponding fusion receptor MFSD-2A suggesting a fusion-permissive environment (Fig. 4a).

Further characterization of the hybrid populations was performed by evaluation of the proliferation rate. Compared to the exponential cell division potential of the parental SK-OV-3 cells only a linear increase in SK-hyb1 cells was observed which continued to proliferate at a low level. In contrast, the cell number of SK-hyb2 cells which was propagated until passage 7, remained nearly unaltered after 72 h indicating little if any proliferative advancement and thus, an eventual cessation of proliferation either due to insufficient growth conditions or post-fusion alteration processes. Together, these data suggested a significantly reduced growth capacity of the ovarian cancer hybrid cell population (Fig. 4b).

Cell cycle analysis demonstrated a pronounced distribution of MSC, SK-OV-3 and SK-hyb1 cells throughout the different cell cycle phases. However, a progressive shift to higher fluorescence intensities was detectable in the different histograms which corresponded to elevated DNA content (Fig. 4c). Compared to a normal diploid set of chromosomes in MSC, the shift to increased DNA content in SK-OV-3 represents aneuploidy which is known for this ovarian cancer cell line displaying a heterogeneous set of chromosomes [34]. Further increase in DNA fluorescence intensity continued in SK-hyb1 cells reaching a level for cells in G_0_/G_1_ nearly to that observed for MSC in G_2_/M phase (Fig. 4c, dotted line). Supportive evidence was obtained from karyotype analysis of SK-hyb1 cells displaying a hypertriploid set of chromosomes. This effect is equivalent to additional chromosomes acquired by SK-hyb1 cells and consequently, supports furthermore the previous cell fusion with MSC.

For in vivo analysis of the proliferative capacity and accompanying tumorigenicity, 4.5 × 10^5^ SK-hyb1 and SK-hyb2 cells were injected subcutaneously into 4 NOD/scid mice, respectively, and tumor development was compared to the application of 4.5 × 10^5^ parental SK-OV-3^GFP^ control cells in 4 NOD/scid mice. Tumor growth of SK-OV-3 cells was detectable already after 20d post injection in (1/4) NODscid mice and was continuously growing. Conversely, no tumor development was detectable in mice transplanted with either SK-hyb1 (0/4) or SK-hyb2 cells (0/4). The average tumor weight induced by SK-OV-3^GFP^ cells reached 161.5 + 110 mg (*n* = 4) after 38d post inoculation and those mice were sacrificed by cervical dislocation according to animal welfare (Fig. 4d). In contrast, application of SK-hyb1 and SK-hyb2 cells was associated with no detectable tumor development at any time and the mice (*n* = 4 for each SK hybrid cell population) subsequently died of age latest after 346d post injection (Fig. 4d).

## Discussion

Previous work has demonstrated that MSC promote enhanced proliferative capacity in different breast and ovarian cancer cells during in vitro co-culture [18, 25]. Likewise, MSC populations also enhanced initial tumor growth of ovarian cancer cells in vivo by displaying a filamentous tumor environment with an increased amount of Ki67-positive cells. These findings were substantiated in co-cultures of ovarian cancer cells with adipose tissue-derived MSC whereby MSC developed altered properties and were therefore characterized as carcinoma-associated (CA-)MSC [23, 35]. Moreover, evidence was presented that distinct bone morphogenic proteins contribute to the capability of CA-MSC to enhance tumor heterogeneity by further promoting tumor growth and by increasing the number of cancer stem cells.

Elevated tumorigenicity in the presence of MSC is also demonstrated by the detection of distant organ metastases in the liver and by a reduced chemosensitivity of explant tumor cells after MSC co-culture when compared to the original SK-OV-3 ovarian cancer cells. MSC-mediated chemoresistance of a drug combination was previously observed in the rare small cell ovarian carcinoma (SCCOHT) [36]. This “chemoresistance” was partially based on tumor-protective effects by enhanced CA-MSC-mediated filament expression including collagen, laminin, elastin, and fibronectin [37] which may explain similar effects in the filamentous tumor microenvironment of MSC co-cultured SK-OV-3 cells.

Increased ovarian tumor heterogeneity is also achieved by fusion of MSC with ovarian cancer cells to generate new hybrid cell populations. Fusion of MSC with cancer cells was previously observed with the MDA-MB-231 breast cancer cell line [26, 27]. Although the resulting fusion populations MDA-hyb1 and MDA-hyb2 demonstrated enhanced tumor growth and elevated formation of metastases compared to the parental MDA-MB-231 cells, these hybrid cells exhibited increased chemosensitivity [27]. Moreover, fusion of MSC with neoplastic MCF10A breast epithelial cells generated hybrid cells which have lost their autonomous growth capacity during post-fusion modifications and underwent aging with cessation of proliferation after a couple of doublings in passage 3 to 4 [28] supporting evidence for anti-tumorigenic effects of MSC during fusion with cancer cells.

Formation of MSC/cancer hybrid cells represents a rare event which can occur by different mechanisms via a certain type of entosis like cancer cell cannibalism [38] or via cell fusion. Previous work suggested the requirement of two coordinated processes for either directed or accidental cell fusion. These include a reorganization of the actin cytoskeleton by different adhesion molecules to generate membrane protrusions with appropriate placement of a transmembrane fusogenic protein [39, 40]. Thus, local membrane protrusions allow the two cell membranes coming into close proximity, whereby such contacts create microdomains that favor exchanges between the adjacent cells. Moreover, certain cell type-specific fusogenic proteins are required, e.g. syncytin-1 and -2 which are predominantly detectable in syncytiotrophoblasts of placenta tissue but also in certain solid tumors [28]. In the course of heterofusion, ASCT-2 (alanine, serine and cysteine selective transporter-2) and MFSD-2A (major facilitator superfamily domain containing 2A) can function as corresponding syncytin receptor on the fusion partner cell [41]. Accordingly, SK-OV-3 cells and the fusion cell partner MSC081113 demonstrated expression of both, syncytin-2 and MFSD-2A suggesting the availability of a fusogenic environment.

The resulting new hybrid cells acquired genomic parts from both parental cells during cell fusion. Consequently, the new karyotype of the fused cell displays more DNA content as compared to the parental cells. Corresponding observations are obtained from SK-hyb1 cell cycle analysis suggesting the acquisition of certain SK-OV-3 and MSC properties. Moreover, the reduced proliferative capacity of the hybrid populations as compared to the parental tumor cell line further supported potential functional alterations mediated by MSC. Together with the observed changes in telomerase, N-, and E-cadherin expression, these data supported a reduced tumorigenicity of the ovarian cancer cell hybrid populations. Indeed, in vivo observations in NOD/scid mice further substantiated the in vitro data demonstrating no detectable tumor development of the SK-hyb1 and SK-hyb2 populations.

In summary, the bipolar MSC functionalities are associated with the all-round supportive regenerative properties and the unique biodiverse capabilities of MSC contributing to increased ovarian cancer growth and potentially enhanced formation of metastases during intercellular communication processes. However, MSC fusion with ovarian cancer cells was associated with the generation of new hybrid cell populations also displaying normal MSC-like properties with reduced proliferative capacity in conjunction with a loss of tumorigenic potential.

## Additional files

Additional file 1: **Figure S1.** Dose-dependent response to carboplatin was tested in SK-OV-3^GFP^ control cells and compared to cells derived from SK-OV-3^GFP^-induced tumor explants and ex vivo tumor cultures from SK-OV-3^GFP^/MSC060616^wt^ co-injections. Relative proliferative capacity was evaluated in a fluoroscan assay following exposure to 1 μM, 10 μM, and 100 μM carboplatin for up to 72 h, respectively. Data represent the mean ± s.d. (*n* = 5) whereby fluorescence values were set to 100% for the corresponding cells in control medium. (TIF 962 kb)

Additional file 2: **Figure S2.** Expression of MSC stem-like markers CD44, CD73, and CD105 was analyzed by RT-PCR in three SK-OV-3^GFP^-derived tumors (mouse tumor 1.1 to 1.3) and SK-OV-3^GFP^/MSC060616^wt^ co-injected tumors (mouse tumor 2.1 to 2.3) as well as in corresponding SK-OV-3^GFP^ tumor explant cultures and in SK-OV-3^GFP^/MSC060616^wt^ tumor explant cultures. GAPDH transcripts served as loading control. (TIF 448 kb)

Additional file 3: **Figure S3.** Hybrid cell formation was observed after fusion of the parental cell populations SK-OV-3^cherry^ P90 and MSC081113^GFP^ P6 by appearance of double-labeled (mcherry and GFP)-expressing yellow fluorescing cells. Separation of this hybrid cell population was performed in two steps by repeated fluorescence-activated cell sorting (FACS). Hybrid cells were collected in microtiter plates with one to two hybrid cells/well and subsequent cell cloning. Two different clones (SK-hyb1 and SK-hyb2) were isolated. (TIF 1151 kb)

## Abbreviations

ASCT-2: Alanine, serine and cysteine selective transporter-2
BRAF: v-Raf murine sarcoma viral oncogene homolog B
CTNNB1: Catenin Beta 1
eGFP: Enhanced green fluorescent protein
epoB: Epothilone B
ERBB2: Erythroblastic oncogene B (=Her2/neu)
FACS: Fluorescence-activated cell sorting
HE: Hematoxylin/eosin
KRAS: Kirsten RAt Sarcoma virus gene
MFSD-2A: Major facilitator superfamily domain 2A
MSC: Mesenchymal stroma/stem cells
PIC3CA: Phosphatidylinositol-4,5-bisphosphate 3-kinase catalytic subunit alpha.
PTEN: Phosphatase and tensin homolog.
STR: Short tandem repeat.
TERT: Telomerase reverse transcriptase.
TP53: Tumor protein 53.
