# Supplementary figures and images for: MSC stimulate ovarian tumor growth during intercellular communication but reduce tumorigenicity after fusion with ovarian cancer cells
Source: Cell Commun Signal. 2018 Oct 13;16:67. doi: 10.1186/s12964-018-0279-1 (PMC6186086; doi:10.1186/s12964-018-0279-1)

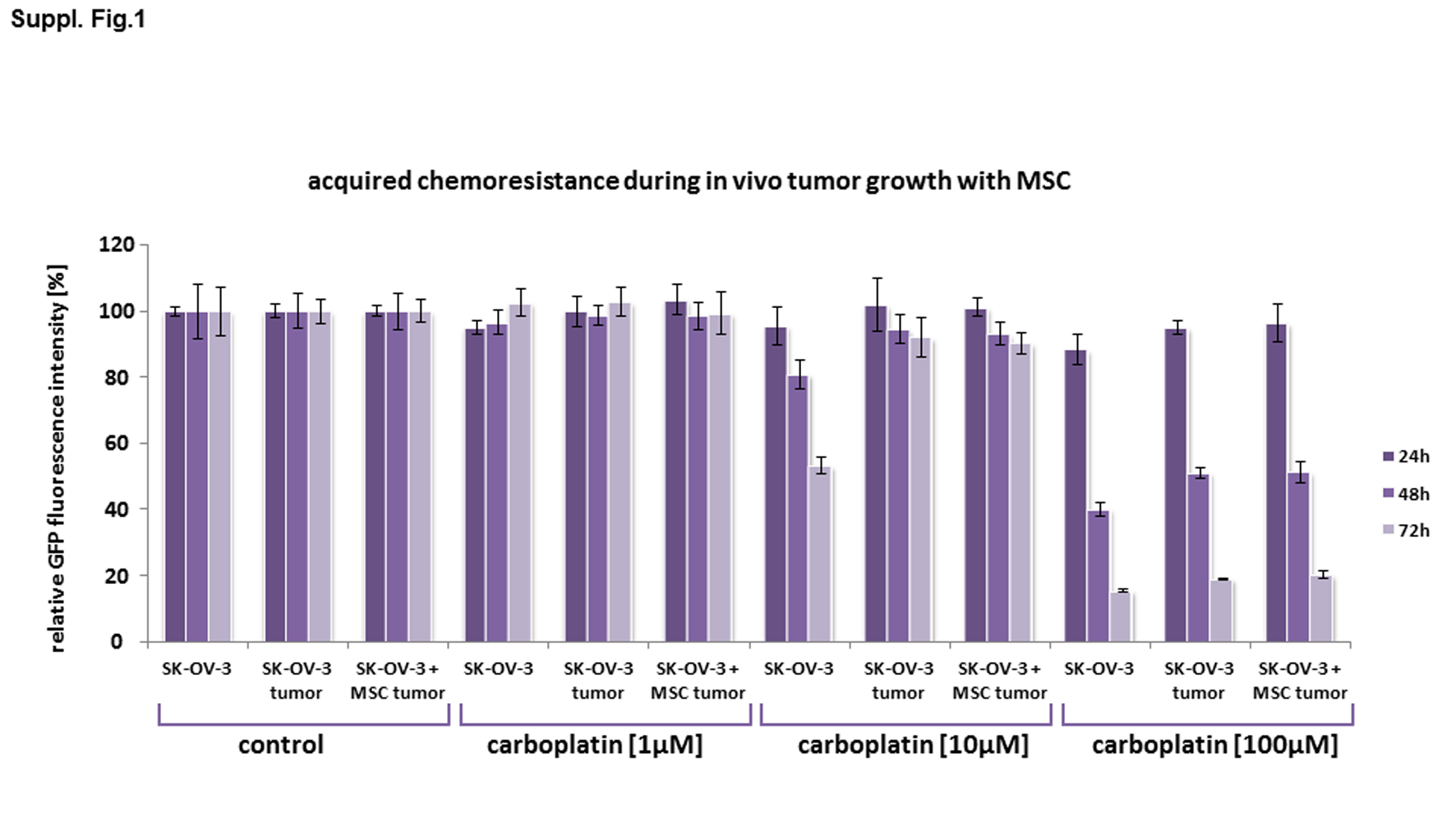

Supplement: Supplementary file 1 — Figure S1. Dose-dependent response to carboplatin was tested in SK-OV-3GFP control cells and compared to cells derived from SK-OV-3GFP-induced tumor explants and ex vivo tumor cultures from SK-OV-3GFP/MSC060616wt co-injections. Relative proliferative capacity was evaluated in a fluoroscan assay following exposure to 1 μM, 10 μM, and 100 μM carboplatin for up to 72 h, respectively. Data represent the mean ± s.d. (n = 5) whereby fluorescence values were set to 100% for the corresponding cells in control medium. (TIF 962 kb) [file 12964_2018_279_MOESM1_ESM.tif]

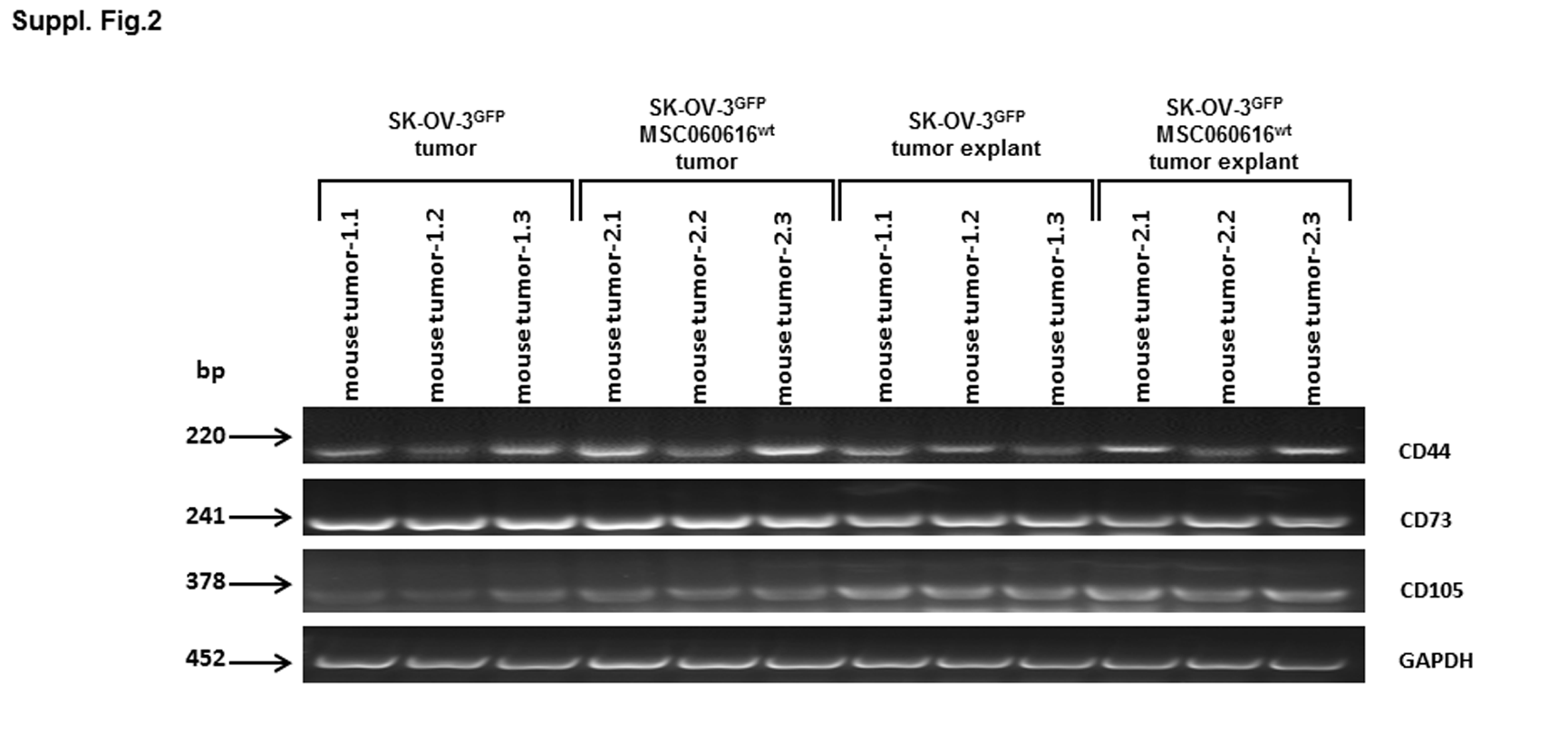

Supplement: Supplementary file 2 — Figure S2. Expression of MSC stem-like markers CD44, CD73, and CD105 was analyzed by RT-PCR in three SK-OV-3GFP-derived tumors (mouse tumor 1.1 to 1.3) and SK-OV-3GFP/MSC060616wt co-injected tumors (mouse tumor 2.1 to 2.3) as well as in corresponding SK-OV-3GFP tumor explant cultures and in SK-OV-3GFP/MSC060616wt tumor explant cultures. GAPDH transcripts served as loading control. (TIF 448 kb) [file 12964_2018_279_MOESM2_ESM.tif]

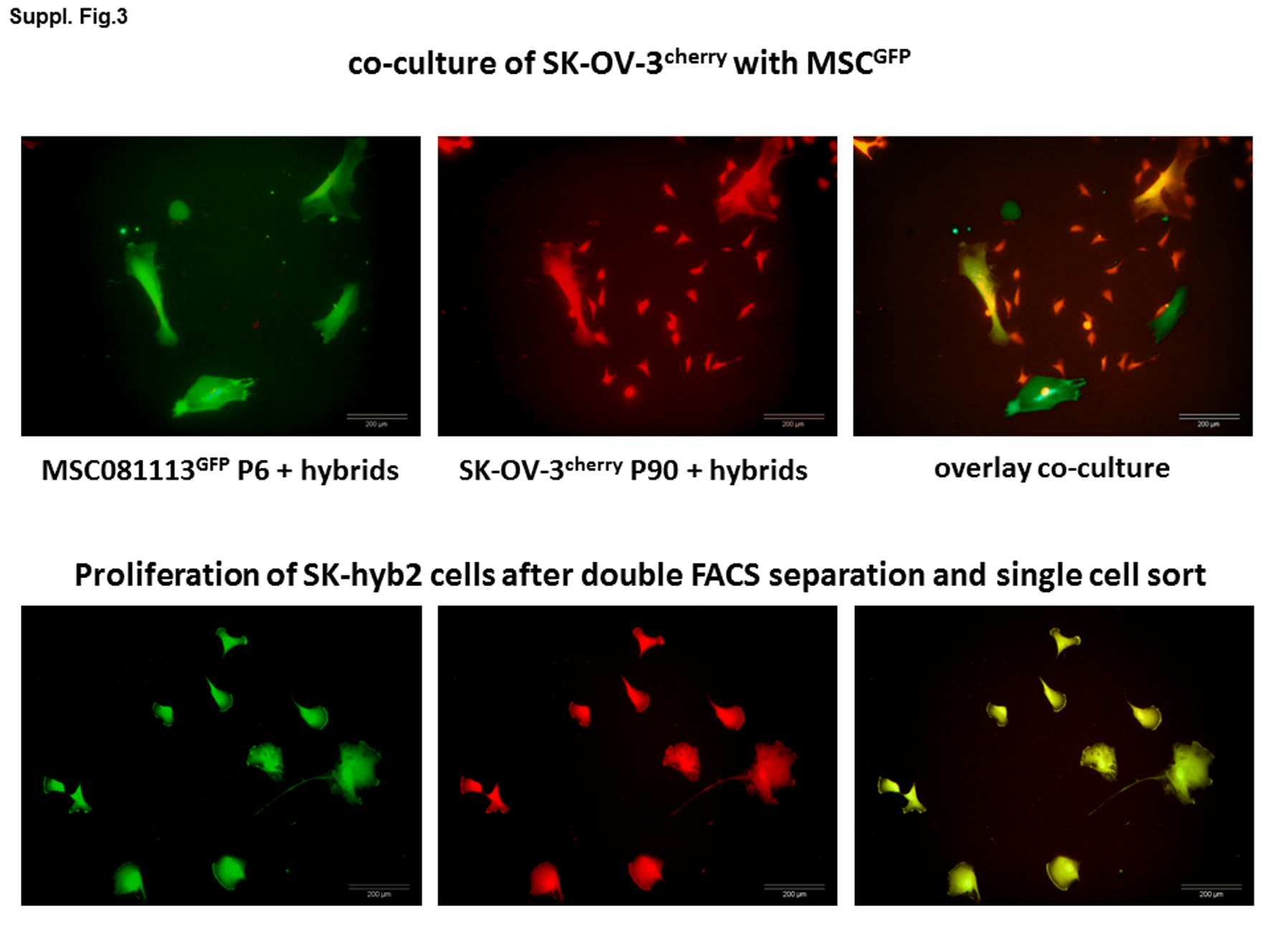

Supplement: Supplementary file 3 — Figure S3. Hybrid cell formation was observed after fusion of the parental cell populations SK-OV-3cherry P90 and MSC081113GFP P6 by appearance of double-labeled (mcherry and GFP)-expressing yellow fluorescing cells. Separation of this hybrid cell population was performed in two steps by repeated fluorescence-activated cell sorting (FACS). Hybrid cells were collected in microtiter plates with one to two hybrid cells/well and subsequent cell cloning. Two different clones (SK-hyb1 and SK-hyb2) were isolated. (TIF 1151 kb) [file 12964_2018_279_MOESM3_ESM.tif]
